# Temporal rate is not a distinct perceptual metric

**DOI:** 10.1038/s41598-020-64984-4

**Published:** 2020-05-26

**Authors:** Aysha Motala, James Heron, Paul V. McGraw, Neil W. Roach, David Whitaker

**Affiliations:** 1 School of Optometry and Vision Sciences, Cardiff University, Cardiff, CF24 4HQ UK; 2 Brain and Mind Institute, Western University, London, ON N6A 5B7 Canada; 3 Bradford School of Optometry and Vision Science, University of Bradford, Bradford, BD7 1DP UK; 4 Visual Neuroscience Group, School of Psychology, University of Nottingham, Nottingham, NG7 2RD UK

**Keywords:** Sensory processing, Human behaviour

## Abstract

Sensory adaptation experiments have revealed the existence of ‘rate after-effects’ - adapting to a relatively fast rate makes an intermediate test rate feel slow, and adapting to a slow rate makes the same moderate test rate feel fast. The present work aims to deconstruct the concept of rate and clarify how exactly the brain processes a regular sequence of sensory signals. We ask whether rate forms a distinct perceptual metric, or whether it is simply the perceptual aggregate of the intervals between its component signals. Subjects were exposed to auditory or visual temporal rates (a ‘slow’ rate of 1.5 Hz and a ‘fast’ rate of 6 Hz), before being tested with single unfilled intervals of varying durations. Results show adapting to a given rate strongly influences the perceived duration of a single empty interval. This effect is robust across both interval reproduction and duration discrimination judgments. These findings challenge our understanding of rate perception. Specifically, they suggest that contrary to some previous assertions, the perception of sequence rate is strongly influenced by the perception of the sequence’s component duration intervals.

## Introduction

Temporal information forms an integral part of human experience. One of the most ubiquitous features of time-varying sensory input is temporal frequency: the rate at which successive signals arrive at our sensory receptor surfaces. In common parlance, this type of information is often referred to as ‘rhythm’, as experienced through virtually all aspects of interaction with our environment including the perception of music and speech. Interactions between movement and the rate of sensory information can result in rhythmic body movement, and this appears to be present even at earliest stages of development^1^. Arguably, rhythm is an inherently intuitive perceptual metric that requires no conscious construction from its multiple temporal component signals. This is evidenced by numerous accounts of unconscious motor actions occurring in line with auditory rhythms^2,3^. Rhythms also provide an element of temporal expectancy and anticipation^4^, as humans quickly become entrained. Moreover, anecdotally, during synchronised tapping to the beat of a song, the global perception of the rate continues to unwaveringly feel unique compared to the individual components which compose that rhythm.

How exactly the brain processes rate across a multitude of timescales and range of sensory inputs is not fully understood. This forms the central question underlying the work presented here, as the perceptual relationship between rate and the component intervals that build those rates is unclear. In a Fourier sense the signal that would be derived from a 333 ms empty interval will possess a commonality with the signal evoked from a 3 Hz rate. Moreover, information regarding one is enough to construct the other, for example, knowing that a rate is presented at a temporal frequency of 6 Hz is enough to deduce single component intervals of 167 ms. However, rate and duration appear phenomenologically distinct. For example, we are able to tap along to a beat without any explicit consideration of the durations between the sequence’s component signals. Furthermore, once a sequence is perceived as having its own rate it becomes harder to break down into its component intervals^5^. This is especially true for higher temporal frequency presentation, as the subjective experience is not of several repeating intervals but of a single metrical construct^5^.

Temporal intervals can be distinguished by whether they are ‘filled’ or ‘empty’. The duration of empty intervals is defined by the temporal gap between two transient sensory signals, such as brief flashes of light or auditory tones. In contrast, filled intervals reflect an ongoing signal presented throughout the period’s duration. Adapting to repeated presentation of filled (continuous) intervals of either auditory or visual stimuli distorts the subjective duration of subsequently presented test stimuli^6^. This duration after-effect is modality specific and shares characteristics with other after-effects, such as those following adaptation to visual motion or orientation^7^, suggesting the existence of bandwidth-limited duration ‘channels’ whose activation level is maximal when test duration coincides with the channel’s preferred duration.

Analogous to this duration after-effect, temporal rate after-effects have been documented in several human adaptation experiments: Specifically, adapting to a relatively fast/slow rate reduces/increases the perceived rate of subsequently presented moderate rate stimuli (respectively)^8,9^. Whilst the modality-specificity of this after-effect has been debated^10^, effects are tuned (bandwidth-limited): beyond a certain limit, large adapt-test differences result in a decay of after-effect magnitude. As with duration after-effects, such observations are consistent with the classical after-effects of visual orientation, size and motion.

The relationship between temporal frequency and duration has received widespread attention. A number of studies suggest that the perceived duration of a stimulus is modified by temporal frequency^11–14^. Specifically, prolonged viewing of visual stimuli with a fixed temporal frequency induces compression in the perceived duration of a subsequently presented test stimulus. It has been suggested that this duration compression occurs even when the perceived temporal frequency of the test stimulus is veridical^15,16^, a finding that suggests temporal frequency and duration interact via a shared mechanism that allows the former to distort the perception of the latter. It has been argued that duration is compressed when temporal frequency adaptation influences the activity of neurons involved in duration encoding, perhaps via modulation of geniculate neuron’s temporal response function^14^ or via slowing the spread of activity within neuronal networks with duration-dependent activation patterns^17,18^.

A common feature of these studies’ experimental design is the use of filled duration adapting stimuli presented for orders of magnitude longer (e.g. 45^18^ or 32 seconds^16^ than the test stimulus (typically around two thirds of a second). At these levels of adapt-test stimulus dissimilarity, the bandwidth-limited nature of duration adaptation effects would predict an absence of interaction between the physical duration of the adaptation stimulus and the perceived duration of the test stimulus. Thus, any apparent interaction between the *duration* information contained within the adapting stimulus and the perceived duration of the test stimulus is likely to escape measurement.

In the current study, we deploy adaptation stimuli comprised of repeatedly presented transients which form sequences of a given temporal frequency. These stimuli contain two key forms of temporal information: sequence temporal frequency and the duration between the sequence’s constituent transients. Both cues are unvarying during the adaptation period, providing trains of adapting durations that were either 166 ms (6 Hz adapting stimuli) or 666 ms (1.5 Hz adapting stimuli). Following adaptation, we then measured the perceived duration of unfilled test durations centred on 333 ms. If the temporal frequency of the adapting stimulus influences perceived duration of the test stimuli this would provide evidence of a shared encoding mechanism. By adapting to temporal frequencies providing adapting durations both shorter *and* longer than test stimulus duration we are able to examine the possibility of *bi-directional* distortions of perceived test stimulus duration. If the adapting sequence’s (local) component durations are being encoded during adaptation (as opposed to (global) rate information alone) repulsion-type duration after-effects will be observed when subjects make duration judgments during the test phase. Alternatively, if component duration information is lost during temporal frequency encoding, test stimulus duration should remain veridical, irrespective of the adapting stimulus’s component durations.

## Results

### Experiment 1: Interval Reproduction

**Figure 1:**
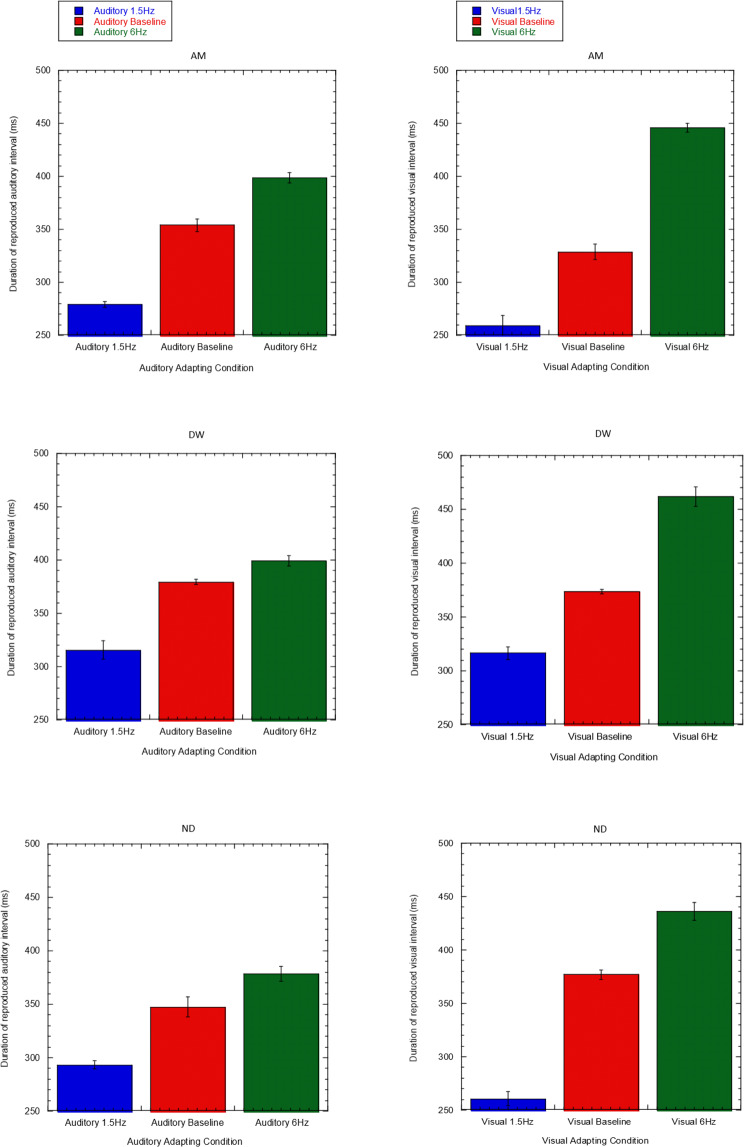
(**a–f**) Reproduced auditory and visual test duration following adaptation to 1.5 Hz and 6 Hz sequences presented within the same modality. Red bars represent baseline duration reproduction values gathered without adaptation. Each row represents a different subject. Values plotted are mean reproduction values of the test interval and error bars indicate standard error derived from the standard deviation across block means. This figure has previously formed part of the doctoral thesis of author A.M^34^.

Figure 1 shows each subject’s overall mean reproduction values for the test interval (333 ms), derived from completing three blocks of 50 trials each. After a period of adapting to a relatively fast (6 Hz) or slow (1.5 Hz) temporal frequency, subjects to reproduced an empty reference interval of 333 ms. Without adaptation, subject AM’s mean reproduced value for the 333 ms auditory test duration (Fig. 1a) was 354 ms. After adapting to a slow rate of 1.5 Hz, reproduced duration for the same stimulus falls to 280 ms. After adapting to a relatively fast rate of 6 Hz, reproduced duration increases to 398 ms. A similar pattern of results is seen in the visual condition (Fig. 1b) and across the other two subjects (Fig. 1c–f).

Paired-samples t-tests (df = 4) were then conducted on duration reproduction data from auditory and visual conditions to test the hypothesis that adapting to the 166 and 666 ms durations embedded within the 1.5 Hz and 6 Hz adapting stimuli caused duration after-effects. Results comparing responses for 1.5 Hz versus 6 Hz adapting stimuli were found to be statistically significant for both visual and auditory conditions (*p* < *0.05)* for each subject (Table 1).

**Table 1:** Results from paired-samples t-tests comparing each condition (Baseline, 1.5 Hz Adapt and 6 Hz Adapt) for auditory and visual conditions for each subject. Results significant at p < 0.05 are denoted with an asterisk (*), and those at p < 0.01 are denoted with a double asterisk (**).

		Auditory	Visual
AM	1.5Hz-6Hz	<0.001**	<0.001**
	Baseline-1.5 Hz	0.001**	0.01*
	Baseline-6Hz	0.005**	<0.001**
DW	1.5Hz-6Hz	0.003**	<0.001**
	Baseline-1.5 Hz	0.004**	0.001**
	Baseline-6Hz	0.007**	0.001**
ND	1.5Hz-6Hz	0.001**	<0.001**
	Baseline-1.5 Hz	0.012*	<0.001**
	Baseline-6Hz	0.004**	0.002**

### Experiment 2: Two-Alternative Forced-Choice (2AFC)

Next, we adopted a criterion-free methodology, asking subjects to make two-alternative forced choice duration discrimination judgments between a 333 ms empty reference interval (presented in either the visual or auditory modality) with a variable duration test interval (presented in the opposite modality to the test stimulus). The proportion of ‘test longer than reference’ responses were plotted as psychometric functions and fitted with a logistic of the form

1 $$y=\frac{100}{1+{e}^{\left(x,-,(,\frac{\alpha }{\theta },)\right)}}$$

where ‘α’ denotes the point of subjective equality (PSE – the 50% response level on the psychometric function representing the physical test duration producing a perceptual match with the 333 ms reference duration) and ‘θ′denotes an estimate of the duration discrimination threshold.

**Figure 2:**
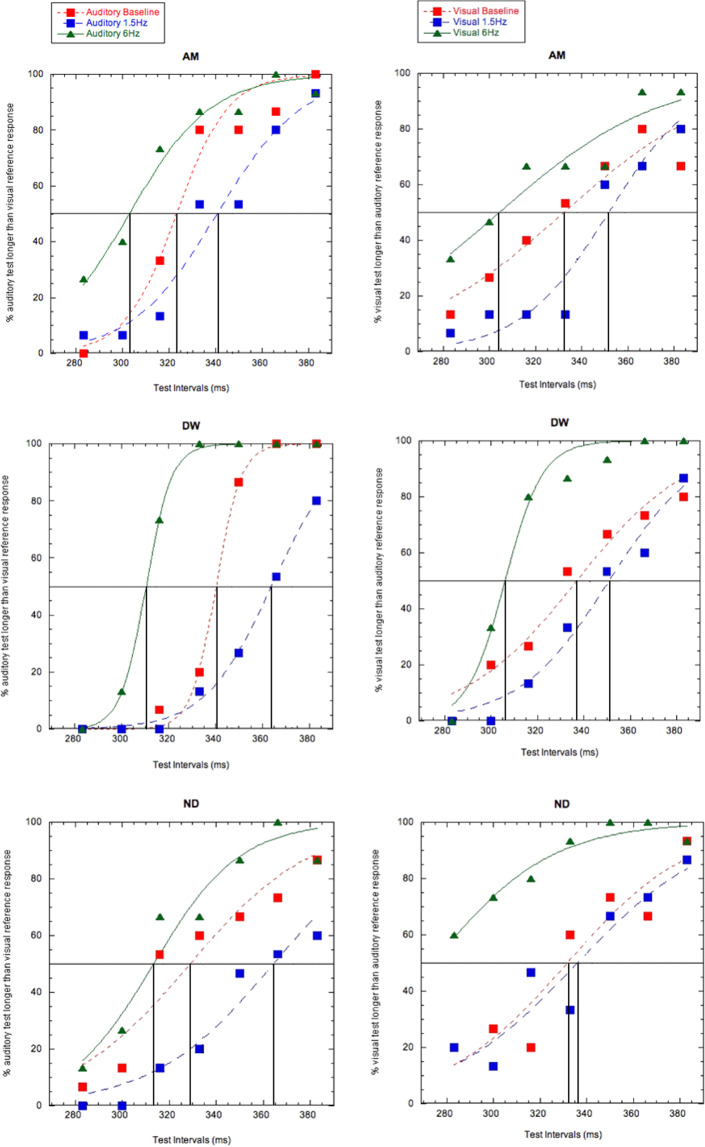
(**a–f**) Auditory-visual unfilled duration discrimination judgments made without adaptation (squares, red data), after adapting to 1.5 Hz stimuli (squares, blue data) and 6 Hz stimuli (triangles, green data). Data are shown for conditions where subjects adapted to auditory (left column) and visual (right column) temporal frequencies. When the adapting stimulus was presented in the auditory modality, reference stimuli were visual and test stimuli were auditory. When the adapting stimulus was visual, reference stimuli were auditory and test stimuli were visual. Horizontal lines indicate the Point of Subjective Equality (PSE) at 50% where subjects were equally likely to respond as the test and reference stimuli equalling the same temporal duration. Vertical lines demonstrate the physical test duration perceptually equating to a 333 ms reference stimulus, for each condition. Data are shown for three different subjects, each occupying a different row. This figure has previously formed part of the doctoral thesis of author A.M^34^.

Results from the auditory temporal frequency adaptation condition (Fig. 2a,c,e) demonstrate induced changes in the perceived duration of the test stimulus. For example, subject AM (Fig. 2a) shows a PSE shifts from 323 ms (no adaptation baseline) to 303 ms or 341 ms after adapting to a relatively slow (1.5 Hz) rate or fast (6 Hz) temporal frequencies, respectively. Thus, adapting to a fast/slow rate expanded/contracted the perceived test interval duration requiring correspondingly shorter/longer unfilled test durations (and thus a smaller PSE value) to maintain perceptual equivalence with the reference stimulus. A similar pattern of effects is observed for the other two subjects (Fig. 2c–f). These distortions of perceived duration are in the opposite direction to the durations adapting stimulus’ component duration and are therefore match the pattern of results observed using duration reproduction (Fig. 1). 2-tailed p-values were obtained via permutation tests comparing PSEs across adapting conditions (Baseline, 1.5 Hz Adapt and 6 Hz Adapt) for each subject. Further details and results of these tests are in the table below (Table 2).

**Table 2:** p-values (2-tailed) obtained via permutation tests comparing PSEs across adapting conditions (Baseline, 1.5 Hz Adapt and 6 Hz Adapt) for each subject. For each test, data was resampled 10,000 times after random shuffling of the adapting condition labels being compared. P-values represent the probability of obtaining an absolute difference in PSEs between conditions equal to or larger than that observed in the original dataset, under the null hypothesis that there is no difference between conditions. Results significant at p < 0.01 are denoted with a double asterisk (**).

		Auditory	Visual
AM	1.5Hz-6Hz	<0.001**	<0.001**
	Baseline-1.5 Hz	0.043*	0.086
	Baseline-6Hz	<0.001**	<0.001**
DW	1.5Hz-6Hz	<0.001**	<0.001**
	Baseline-1.5 Hz	<0.001**	0.085
	Baseline-6Hz	<0.001**	<0.001**
ND	1.5Hz-6Hz	<0.001**	0.005**
	Baseline-1.5 Hz	<0.001**	0.713
	Baseline-6Hz	0.067	0.001**

A further notable feature of Fig. 2’s effects is the relative lack of transfer of any adaptation effects from the adapting stimulus to the reference stimulus. Had this transfer taken place, test and reference stimuli would both have undergone perceptual distortion in the same direction, with the effect that judgments about the relative durations of these two stimuli would have appeared similar in the baseline and adapted conditions. Whilst some partial transfer cannot be conclusively ruled out, the clear PSE shifts shown in Fig. 2 demonstrate that adaptation had a stronger effect on the test stimulus than the reference, suggesting that the mechanisms driving adaptation are sensitive to the sensory modality within which the adapting stimuli are presented.

### Experiment 3: Filled Interval Control

Despite the explicit nature of the duration judgments being made (whether reproduction or 2AFC), one possible explanation for our effects is that subjects may have compared the temporal frequency of the adapting and test stimuli. This is perhaps more likely when (unfilled) durations embedded in the adapting stimulus shared phenomenological similarity with the (unfilled) test durations. To test this possibility we conducted a further experiment where temporal frequency adaptation was followed by reproduction of a *filled* test duration.

**Figure 3:**
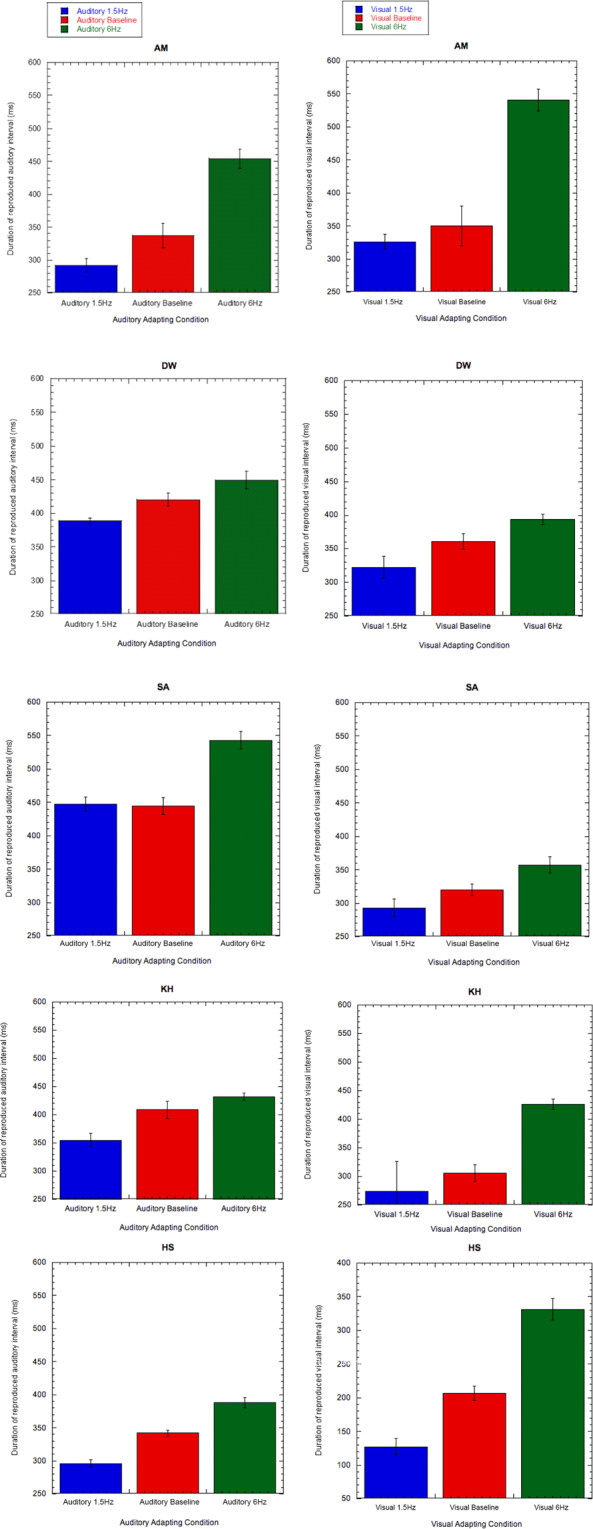
(**a–j**) The after-effect of adapting to different temporal rates of 1.5 Hz and 6 Hz demonstrated through interval reproduction for auditory and visual filled interval conditions. Red bars represent baseline duration reproduction responses gathered without adaptation. Each row represents a different subject. Values plotted are mean reproduction values of the test interval and error bars indicate standard error. A version of this figure has previously formed part of the doctoral thesis of author A.M^34^.

Results from this experiment demonstrate similar after-effects to those observed in Experiment 1. Specifically, for subject AM (Fig. 3a,b), adapting to a slow auditory frequency results in a contraction of the test interval (from around 337 ms to 292 ms). Whereas adapting to a faster auditory frequency results in the opposite (a shift from 337 ms to 456ms). Similarly, in vision, the same subject reports a subjective contraction (from 350ms to 326 ms) after adapting to a slow visual rhythm, whereas adapting to a fast visual rhythm results in an expansion of the test interval (from 350 ms to 540 ms). These results are replicated in subject DW for auditory and visual conditions, and also in the visual condition for subject SA. Despite no significant difference being observed between auditory baseline and 1.5 Hz conditions for subject SA, a similar pattern is observed in subject SA across the auditory baseline and 6 Hz condition (and also between 1.5 Hz and 6 Hz). The pattern of results from this experiment verify those of experiments 1 and 2 and demonstrate that the ability of human subjects to adapt to various unimodal rhythms can be demonstrated in the subsequent perception of both empty *and* filled intervals. As before, paired-samples t-tests were conducted to compare filled duration reproduction across adapting conditions. As was the case in Experiment 1, significant differences between 1.5 Hz and 6 Hz adapting conditions were consistently found across all subjects (Table 3).

**Table 3:** Results from paired-samples t-tests comparing each condition (Baseline, 1.5 Hz Adapt and 6 Hz Adapt) for auditory and visual conditions for each subject. Results significant at p < 0.05 are denoted with an asterisk (*), and those at p < 0.01 are denoted with a double asterisk (**).

		Auditory	Visual
HS	1.5Hz-6Hz	<0.001**	<0.001**
	Baseline-1.5 Hz	<0.001**	<0.001**
	Baseline-6Hz	0.001**	<0.001**
AM	1.5Hz-6Hz	<0.001**	<0.001**
	Baseline-1.5 Hz	0.205	0.468
	Baseline-6Hz	0.003**	<0.001**
DW	1.5Hz-6Hz	0.009**	0.012*
	Baseline-1.5 Hz	0.074	0.166
	Baseline-6Hz	0.194	0.08
SA	1.5Hz-6Hz	<0.001**	0.007**
	Baseline-1.5 Hz	0.848	0.024*
	Baseline-6Hz	<0.001**	0.035*
KH	1.5Hz-6Hz	<0.001**	0.021*
	Baseline-1.5 Hz	0.008**	0.577
	Baseline-6Hz	0.14	<0.001**

## Discussion

The central question underpinning our experiments is whether rate forms a distinct perceptual metric, or whether it is processed as an aggregate of its component durations. The present experiments employ methods of temporal reproduction and 2AFC temporal comparison and demonstrate that adapting to a particular temporal frequency markedly distorts the perception of a single empty interval. These types of after-effects persist even when the test interval is a filled signal. Furthermore, this effect is bi-directional: the unfilled *and* filled durations can be perceptually expanded or contracted via adaptation to 1.5 Hz and 6 Hz sequences. These findings question our understanding of rate adaptation and suggest that adaptive after-effects as a result of prolonged exposure to a temporal rate may actually be a result of distortions in the interval durations composing those temporal rates. A parsimonious view would be that the rate after-effect may be a direct consequence of the established duration after-effect^6^, and that current models of temporal duration estimation can be extended to models of temporal rate.

Such a viewpoint is inherently contentious, since it contradicts our natural appreciation of rate. We don’t experience any conscious effort in combining individual intervals to form the global percept of rate. But this lack of consciousness of any reconstructive process may well reflect the ultimate goal of our sensory system – a relatively rapid and reliable appreciation of the physical world around us. Just as our visual appreciation of a house would fundamentally lack efficiency were it to entail the systematic piecing-together of each constituent brick, so our perception of sensory rate is better accomplished without the need to bring repeated judgements of temporal intervals to our conscious attention. At the level of the early visual cortex, visual processing involves a patchwork array of neural mechanisms each devoted to a local analysis of luminance and chromaticity. Yet our visual percept of the world around us is not one of a jigsaw that needs piecing together, but of a smooth, global construct. This perceptual outcome is very much dependent upon the extremely lawful spatial arrangement of the constituent local mechanisms – when this reliability breaks down, such as in amblyopia or retinal disease, severe consequences for global visual performance result. But in normal sensory systems, provided higher levels of analysis ‘understand’ what the lower levels of input represent, then they can be reconstructed successfully, and most important, unconsciously.

Our findings reinforce the sensory-specificity of after-effects as a result of adapting to empty intervals of time. Recall that in our 2AFC experiment, judgements were made of the duration of an adapted sense relative to a ‘standard’ presented in a different sense. As noted by Heron *et al*.^6^ in an investigation of filled intervals, if the effects of adapting to empty intervals transferred cross-modally between test and reference stimuli, then both the reference and test should become simultaneously distorted; thus resulting in diminished (if any) after-effects. In their work, psychophysical after-effects to filled durations prevail in two-alternative forced-choice tasks despite the reference interval being presented within a different modality to the one adapted to. Here, we demonstrate that similar after-effects persist, and are consistent with those observed in the absence of reference stimuli, as gathered from our interval reproduction experiments.

Previous studies have presented a difference in modality specificity between rate and duration. Specifically, it has been suggested that the perception of duration is sensory-specific^6^ whereas rate has been shown to be processed cross-modally^8^. Becker and Rasmussen^9^ adapted participants to unimodal auditory and visual rates and found evidence for rate after-effects. These effects however, were only manifest when adapting and test stimuli were presented within the same sensory modality. These findings have also been replicated within the tactile modality^10^. Cross-modal cue integration of rate and/or duration must therefore be enacted at a processing stage downstream of sensory-specific adaptation effects^19^. It may be asked why this experiment was not conducted in reverse, i.e. why empty intervals were not adapted to and tested with rate sequences? An inherent complication with this approach, however, is perceptual similarity between unfilled adapting durations and their interstimulus intervals. An alternative approach would be adapting to filled durations then testing perceived rate. Relatedly, Heron *et al*.^6^, varied the inter-stimulus interval between filled adapting durations to create conditions of matched visual adapting duration (160 ms) but different adapting temporal frequencies (1.1 Hz vs 0.72 Hz). Duration aftereffects were consistent with the adapting stimulus duration and were found to be invariant across the two adapting temporal frequencies. An interesting avenue for further experiments would be to repeat this experiment but change the test phase judgment to one of temporal frequency.

Pashler^20^ attempted to use measures of temporal precision (as opposed to our examination of temporal distortion) to evaluate whether the timing of short auditory intervals is mediated by interval-based or beat-based timers^20^. In one of two experiments, subjects were exposed to a sequence of uniform tones, followed by a 2AFC method similar to ours. In a second experiment, Pashler had subjects reproduce two test tones via 2 keypress responses. Pashler asserts that if improved performance in such tasks required beat-based mechanisms, performance should also be most precise in those conditions where the interval between the standard and test consistently matched the standard interval – a result that was not found. Grahn^21^ suggests that in tasks assessing beat-based timers, it may be possible for these mechanisms to exist but for performance to remain unaffected, further suggesting that a lack of performance improvement through beat-based timers does not preclude the possibility that they still exist or that they may have been implicitly active at the time. Grahn and Brett^22^ investigated this further in an fMRI paradigm. Specifically, they found that when subjects reproduced more complex rates i.e. rates composed of multiple interval lengths, rates that were specifically designed to give rise to a beat were reproduced more accurately than those rates that did not invoke subjective beat perception^22^. The authors question whether it is perhaps possible that beat-based mechanisms only present measurable differences in more complex timing sequences and behaviour^21,22^. Whilst this notion appears plausible, it still poses the question of why such a mechanism exists, if not to improve performance in all instances where it can – why would such a mechanism lie dormant even in instances where it is able to influence behaviour, for example, in the instance of Pashler’s aforementioned first experiment^20^? What is the evolutionary advantage? The fact that we explored this further using psychophysical adaptation techniques and still failed to observe explicit behavioural evidence of a beat-based timer further adds to this contention. Furthermore, any beat-based timing mechanism would have to prevail in addition to, and alongside an interval timing mechanism, as many events in real life have to be timed without the structural frame of rates^21^.

Literature concerned with examining the perception of isochronous (regularly paced) against anisochronous (irregularly paced) temporal patterns has, on numerous occasions, suggested that discrimination of anisochronous sequences is significantly worse compared to performance for isochronous sequences^23–27^. Evidence gathered from the present set of experiments suggests that rate may not be a distinctly independent temporal feature and instead may be processed as repeatedly presented single intervals of time. These findings are neatly able to add to the body of literature being gathered on anisochrony perception and allow for another explanation of poorer performance for irregular sequences. Since rate as a feature is partially processed on an interval-by-interval basis, an anisochronous sequence thus fails to allow a consistent representation of an internal mean and instead is processed as a collection of successive, yet unrelated intervals, thereby partially explaining poorer performance with irregular compared to regular sequences.

Despite distinct differences between a simple rate and a more complex ‘beat’, the results presented here are supported by studies exploring related distinctions using neuroimaging. In efforts to deduce the differences in beat-based and duration-based auditory timing, Teki and colleagues^28^, assessed a dissociation of the cortical networks mediating these two timing constructs. Sequences of irregular intervals were structured in such a way to make encoding of their component intervals more likely, subsequently recruiting duration-based neural networks. In contrast, regular sequences would recruit beat-based timing mechanisms to calculate the regularly repeated (and uniform) intervals. The contributions in neural circuitry were found to be clearly dissociated for the two sequences. Specifically, duration-based timing was mediated by the olivocerebellar network employing the cerebellum and inferior olive whereas beat-based timing activated a striatio-thalamo-cortical network including the putamen, caudate, thalamus, pre-supplementary motor area/supplementary motor area, premotor and dorsolateral prefrontal cortex^28^. Later evidence from Teki *et al*.^29^ suggests a high-level of co-dependence between these networks, implicating the interconnected nature of these networks not only anatomically, through the cerebral cortex and numerous synaptic pathways^29^, but also functionally.

In conclusion, using two different methodologies we have demonstrated rate adaptation strongly affects the perception of subsequently presented durations. We suggest that the distortions which can be imposed upon rate through sensory adaptation, are in fact a consequence of adaptation to the component intervals that cumulatively compose the perception of a rate sequence.

## Methods

### Subjects

6 subjects (1 female, 5 male) participated (mean age = 27, sd = 13.2 years), with normal hearing and visual abilities. Two participants had previous experience of psychophysical data collection (authors AM & DW), whereas the other participants (ND, SA, KH & HS) were completely naïve to both psychophysics and also the purpose of the experiments. The experiments received ethical approval from the Research Ethics Committee at the School of Optometry and Vision Sciences, Cardiff University (U.K.), and all experiments were performed in accordance with relevant guidelines and regulations. Written informed consent was obtained to participation.

### Apparatus & stimuli

Brief visual or auditory stimuli were presented. Stimulus generation and presentation was controlled by an Intel® Core™ i5-4460 desktop computer running Microsoft Windows 7. Experiments were programmed in MATLAB 8.6 (Mathworks, USA) in combination with Psychophysics Toolbox 3 (http://www.psychtoolbox.org). Stimulus timing was verified using a dual-channel oscilloscope.

### Visual

Visual stimuli were presented on an Eizo EV2436W monitor. These were bright (274 cd/m^2^) white circular flashes presented centrally against a uniform dark background (0.32 cd/m^2^). Stimulus duration was a single frame (approximately 16 ms at the monitor frame rate of 60 Hz). At the viewing distance of 60 cm the circular flash subtended a diameter of approximately 10.5° of visual angle.

### Auditory

Auditory stimuli consisted of brief (16 ms duration) bursts of white noise generated by a Xonar Essence STX (ASUS) soundcard (https://www.asus.com/us/Sound-Cards/Xonar_Essence_STX/) with a sampling rate of 44,100 Hz. Stimuli were delivered using Sennheiser HD280 Pro Headphones at an SPL of 70 dB. Auditory stimuli were specifically chosen to be lacking in any possible pitch, timbre, or dynamic variations to avoid confounding influences on rate^30^.

### Control filled experiment

One subject who participated in Experiment Three was run in a different location due to the experimenter moving labs. The same scripts were used and all experimental conditions were kept identical to the earlier set of participants however, some of the experimental apparatus varied. Specifically, stimulus generation and presentation was controlled by a Lenovo Thinkpad laptop with Intel® Core™ i7-5500U running Microsoft Windows 7. Auditory stimuli were presented using a Steinberg UR22 soundcard with a sampling rate of 44,100 Hz. Stimuli were delivered using Sennheiser HD 205 headphones at an SPL of 60-70db. All stimuli remained consistent with the original experiments and were supra-threshold.

### Procedure

**Figure 4:**
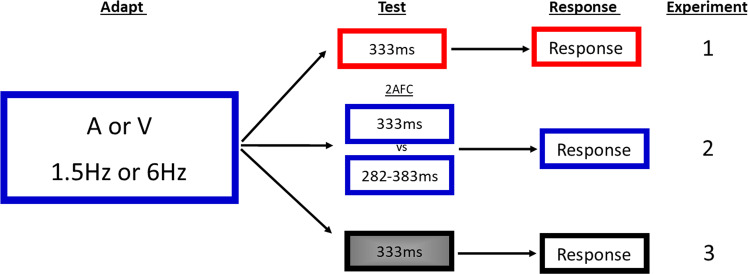
Schematic depiction of the three experiments subjects completed. The numbers on the right of the schematic represent the experiment. All subjects were presented with an adaptation period to either a slow (1.5 Hz) or fast (6 Hz) unimodal rate (either auditory or visual). Depending on the task they were then presented with a range of different test stimuli, followed by a response period. In experiment 1, subjects were presented with an empty 333 ms interval to the same modality that they had recently adapted to. The task then required subjects to reproduce this empty interval by tapping on a response disk. The same design was adapted to 2AFC in experiment 2, and the test interval was adapted to a filled interval in experiment 3. See text for further detail.

The specificity of rate adaptation and temporal after-effects were investigated by adapting subjects to temporal rates (either 1.5 Hz or 6 Hz, fixed within a block) and testing with single, empty intervals using both interval reproduction and two-alternative forced choice methods. The interval reproduction method requires subjects to recreate their internally perceived durations after adaptation and therefore provides a very explicit response. Despite being an explicit measure of temporal perception, interval reproduction methods have been criticised for being subject to criterion-dependent bias^31^. This method was therefore used alongside a less criterion-dependent two-alternative forced-choice (2AFC) duration discrimination task. Here, an unfilled reference interval was presented to the non-adapted modality (e.g., audition) followed by a variable (282–383 ms in six linear steps) unfilled test interval presented to the opposite (adapted) modality (e.g. vision). The subject’s task was to report (via key press) whether the test stimulus was shorter or longer than the reference stimulus. Following initial practice sessions, a process of data collection (lasting approximately 6 hours) began in a series of sessions spread over several days (Fig. 4).

### Testing

#### Experiment 1: Interval Reproduction

The interval reproduction experiment began with a 10 second adaptation phase on every trial where a train of stimuli with a fixed rate was presented to subjects. The sensory modality (either auditory clicks or visual flashes) and presentation rate (either 1.5 Hz or 6 Hz rate) of the adapting stimuli was held constant within an experimental session. The adaptation phase was followed by a test period composed of an empty reference interval of 333 ms presented within the adapted modality. The test interval was identical on each trial. Subjects then reproduced this empty interval by tapping twice on the response disk (a piezoelectric transducer) used to record interval reproduction^32^. The resulting voltage output was fed to the ‘audio in’ of the soundcard as a recording which was analysed within MATLAB to extract the duration of the reproduced interval. The transducer was enclosed in a sound-dampening environment and shielded from sight of the subject. To further eliminate the possibility of auditory feedback, white noise was played via the headphones throughout the tapping response phase^33^. The interval between the adaptation and test phase was set to 500 ms as was the inter-trial interval. Each subject completed 50 trials per each of the three conditions, and the values plotted are an average of these data (150 trials per each sensory modality). Outliers (responses with a standard deviation of more than 60 ms - roughly 20% of the reference interval) were excluded from analysis. Conditions were counterbalanced across subjects.

#### Experiment 2: Two-Alternative Forced-Choice (2AFC)

The 2AFC procedure was identical to the interval reproduction however, following the adaptation sequence, a reference interval of 333 ms was presented in the modality not adapted to, followed by a test interval presented in the same modality adapted to (randomly ranging 282–383 ms in six linear steps, centred around 333 ms). The response period required subjects to respond to whether the test stimulus was shorter or longer than the reference stimulus via a keypress. The interval between the adaptation and test phase was set to 500 ms, and the inter-trial interval was randomly set to 500ms-1 second. Each test interval was presented at least 15 times per condition, per subject, resulting in a total of 105 total trials per each condition, and 315 per sensory modality. The randomly selected test interval was always presented after the reference interval. Conditions were counterbalanced across subjects.

Performance feedback was not provided during either task. Baseline data, collected prior to adaptation were gathered for all conditions.

#### Experiment 3: Filled Interval Control

This experiment was a control variation with all experimental details and procedure being identical to experiment 1 with the exception of the test stimulus, which was adapted from what was previously an empty interval to continuous stimulation of either white noise or a white circular flash via a filled interval of the same temporal duration (333 ms). Subjects then reproduced this (filled) interval by depressing a key on a keyboard to mark the duration of the filled test interval. The interval between the adapting and test phase was set to 500 ms, as was the interval after the presentation of the test interval. Each of the reproduction values plotted in Fig. 3 are an average of 100 trials per condition, per subject (300 per sensory modality). Conditions were counterbalanced across subjects.
