# Publisher Correction: Temporal rate is not a distinct perceptual metric

**DOI:** 10.1038/s41598-020-68539-5

**Published:** 2020-07-07

**Authors:** Aysha Motala, James Heron, Paul V. McGraw, Neil W. Roach, David Whitaker

**Affiliations:** 1 School of Optometry and Vision Sciences, Cardiff University, Cardiff, CF24 4HQ UK; 2 Brain and Mind Institute, Western University, London, ON N6A 5B7 Canada; 3 Bradford School of Optometry and Vision Science, University of Bradford, Bradford, BD7 1DP UK; 4 Visual Neuroscience Group, School of Psychology, University of Nottingham, Nottingham, NG7 2RD UK

Correction to: *Scientific Reports* https://doi.org/10.1038/s41598-020-64984-4, published online 26 May 2020

The original HTML version of this Article contained a typographical error in Equation 1.

$$y= \frac{100}{1+ {e}^{\left(x,-,(,\frac{a}{\theta },)\right)}}$$

now reads:

$$y= \frac{100}{1+ {e}^{\left(x-(\frac{a}{\theta })\right)}}$$

This error has now been corrected in the HTML version; the PDF version was correct from the time of publication.

